# Complete percutaneous angio-guided approach using preclosing for venoarterial extracorporeal membrane oxygenation implantation and explantation in patients with refractory cardiogenic shock or cardiac arrest

**DOI:** 10.1186/s13054-021-03522-8

**Published:** 2021-03-07

**Authors:** Anne-Sophie Martin-Tuffreau, François Bagate, Madjid Boukantar, Gabriel Saiydoun, Andrea Mangiameli, Laura Rostain, Gauthier Mouillet, Antonio Fiore, Olivier Langeron, Armand Mekontso-Dessap, Nicolas Mongardon, Thierry Folliguet, Emmanuel Teiger, Romain Gallet

**Affiliations:** 1 Service de Cardiologie, APHP, Hôpitaux Universitaires Henri Mondor, 41 avenue du Maréchal de Lattre de Tassigny, 94000 Créteil, France; 2 AP-HP, Hôpitaux Universitaires Henri-Mondor, Service de Médecine Intensive Réanimation, 94010 Créteil, France; 3 Univ Paris Est Créteil, CARMAS, 94010 Créteil, France; 4 Univ Paris Est Créteil, INSERM, IMRB, 94010 Créteil, France; 5 Department of Cardiac Surgery, APHP, Hôpitaux Universitaires Henri Mondor, 94010 Créteil, France; 6 Service d’anesthésie-réanimation chirurgicale, DMU CARE, DHU A-TVB, Assistance Publique-Hôpitaux de Paris (AP-HP), Hôpitaux Universitaires Henri Mondor, 94010 Créteil, France; 7 U955-IMRB, Equipe 03, Inserm, Univ Paris Est Creteil (UPEC), Ecole Nationale Vétérinaire D’Alfort (EnVA), 94700 Maisons-Alfort, France

**Keywords:** ECMO, Percutaneous cannulation, Closure device, Cardiac arrest, Cardiogenic shock

## Abstract

**Background:**

The approach for veno-arterial extracorporeal membrane oxygenation implantation (VA-ECMO) in patients with cardiogenic shock can be either surgical or percutaneous. Complete angio-guided percutaneous implantation and explantation could decrease vascular complications. We sought to describe the initial results of complete percutaneous angio-guided ECMO implantation and explantation using preclosing.

**Methods:**

All consecutive patients who underwent peripheral femoro-femoral VA-ECMO percutaneous implantation for refractory cardiogenic shock or cardiac arrest were enrolled in a prospective registry (03/2018–12/2020). Percutaneous preclosing using two closing devices (Perclose ProGlide, Abbott) inserted before cannulation was used in both femoral artery and vein. Explantation was performed using a crossover technique under angiographic guidance. The occurrence of vascular complication was recorded.

**Results:**

Among the 56 patients who underwent percutaneous VA-ECMO implantation for cardiogenic shock or refractory cardiac arrest, 41 underwent preclosing. Femoral vessel cannulation was successful in all patients and total cannulation time was 20 (10–40) min. Weaning from ECMO was possible in 22/41 patients (54%) and 12 (29%) patients were alive at day 30. Significant vascular complications occurred in 2/41 patients. Percutaneous decannulation was performed in 20 patients with 19/20 technical success rate. All femoral arteries and veins were properly closed using the pre-closing devices without bleeding on the angiographic control except for one patient in whom surgical closure of the artery was required. No patient required transfusion for access related significant bleeding and no other vascular complication occurred. Furthermore, no groin infection was observed after full percutaneous implantation and removal of ECMO.

**Conclusion:**

Emergent complete percutaneous angio-guided VA-ECMO implantation and explantation using pre-closing technique can be an attractive strategy in patients referred for refractory cardiogenic shock.

**Supplementary Information:**

The online version contains supplementary material available at 10.1186/s13054-021-03522-8.

## Introduction

Cardiogenic shock is a dramatic complication of both ischemic and non-ischemic heart failure. Its outcome is poor, with a mortality rate of 30–60% depending on the etiology [1–3]. The initial treatment relies on inotropic therapy to increase the cardiac output [4–6]. However, this treatment will fail in a non-negligible number of patients, leading to refractory cardiogenic shock. Veno-arterial extracorporeal membrane oxygenation (VA-ECMO) has been described as an attractive strategy for the support of cardiogenic shock but also for the support of cardiac arrest [7–9]. It can be implanted trough surgical or percutaneous approach [10, 11]. Due to the systematic use of echo-guiding for cannula insertion and to the improvement of techniques, percutaneous approach is increasingly used for cannulation and may be associated with a lower risk of vascular complication, namely limb ischemia or hematoma [11]. Angiographic guidance helps to monitor wires and cannulas progression in the vessels and might further increase the success rate and the safety of implantation.

Regarding removal, surgical femoral cutdown is considered as standard practice but frequently associated with severe complications such as hemorrhage, delayed wound healing and infections [11, 12]. Other techniques such as manual compression or post-closing using closing devices have been described but none of these are ideal [10, 11, 13–15]. Hence, decannulation remains at risks of complications because of the lack of standardized approach. The preclosing technique using Perclose ProGlide^®^ (Abbott^®^ Chicago, Illinois, United States), has been widely used for closing in endovascular procedures, with similar complications rate as compared to surgical access and closure [16]. In transcatheter aortic valve replacement (TAVR), preclosing is usually combined with a routine crossover technique to ensure the proper closure of the access site [17]. It provides a high success rate and is associated with a significant reduction in major vascular and bleeding complications. This preclosing and crossover technique may enable a complete percutaneous approach for VA-ECMO implantation and explantation which could decrease the risk of vascular complications in these high-risk patients.

Thus, we aimed to describe the initial results of a complete percutaneous angio-guided approach using preclosing for ECMO implantation and explantation in patients referred for cardiogenic shock or cardiac arrest.

## Methods

### Study design and population

All consecutive patients admitted at Henri-Mondor University Hospital (Créteil France) and supported with percutaneous VA-ECMO for refractory cardiac arrest or refractory cardiogenic shock from March 2018 to December 2020 were included in a monocentric prospective observational registry. All patients’ data and outcomes were analyzed with a special focus on the subgroup of patients who underwent a preclosing procedure.

Study protocol was approved by Henri Mondor University Hospital’s ethics committee (registration code n°1778041).

### Percutaneous preclosing and angio-guided cannulation procedures

ECMO was implanted in the cardiac catheterization laboratory under angiographic guidance for all patients. All procedures were performed by senior interventional cardiologists trained to echo-guided vascular puncture, preclosing and large bore access. All patients were mechanically ventilated and sedated except two, in whom sedation and local anesthesia were performed.

The common femoral artery and femoral vein were punctured under ultrasound guidance using the Seldinger’s technique. The position of the wires (in the aortic root and the superior vena cava) was verified using angiography. A preclosing technique using two Perclose ProGlides^®^ (Abbott^®^, Chicago, Illinois, USA) was performed before cannulation in both the femoral artery and the femoral vein, as previously described [18]. Then, after preparation with adequate dilatators, femoral vessels were cannulated over a stiff wire (Amplatz Super Stiff^®^, Boston Scientific^®^, Marlborough, Massachusetts, USA) under angiographic guidance to confirm the positioning of the wires and the progression of the cannulas. A 23 cm length 17–19 Fr arterial cannula (Maquet^®^ Rastatt, Germany) (17 or 19 Fr depending on the patient’s morphology) was inserted in the common femoral artery and a 55 cm 23 Fr venous cannula (Maquet^®^, Rastatt, Germany) was inserted into the femoral vein. The venous cannula was advanced to the right atrium under angiographic guidance. An additional anterograde perfusion line (6 Fr sheath, Terumo^®^, Shibuya, Tokyo, Japan) was systematically inserted into the superficial femoral artery under ultrasound guidance (usually before the insertion of the arterial cannula) and “y” connected to the arterial cannula to reduce the risk of limb ischemia [19]. The ProGlides^®^ threads were carefully separated, coiled around a compress and an occlusive dressing was made. In refractory cardiac arrest, the cannulation was organized to be as fast as possible; therefore, as soon as the ECMO circuit was primed, the cannulas were inserted and the ECMO started. In those patients, distal perfusion line insertion and preclosing could be performed during priming but in a few patients (mostly the first ones), preclosing was not performed and distal perfusion line was inserted after ECMO initiation.

Cannulation time was defined as the time between the first puncture and the start of ECMO support.

### Angio-guided percutaneous decannulation procedure

Once ECMO removal criteria were fulfilled in the intensive care unit, decannulation was performed percutaneously in the cardiac catheterization laboratory under angiographic guidance. After switching the ECMO off and clamping the lines, a 14- or 16-Fr dilator (fitting the internal diameter of the 17-Fr or 19-Fr arterial cannula) was inserted with a 0.035″ J-shaped Starter guidewire (Boston Scientific^®^, Marlborough, Massachusetts, United States). The contralateral common femoral artery was punctured and the crossover balloon occlusion technique was used to enable a safe decannulation as previously described [17]; briefly, a peripheral balloon (8–12 mm diameter depending on common femoral or external iliac size) was inserted through a crossover sheath and inflated at low pressures (0.5 atm) in the common femoral or external iliac artery to allow a non-traumatic occlusion of the vessel. Then, the arterial cannula was removed and the knots were tightened. Balloon occlusion allowed the operator to tight the knots with the knot pusher without bleeding. The balloon was then deflated and an angiography was performed through the crossover sheath to ensure the proper closing of the artery and the absence of complications. In case of incomplete closure, and depending on the importance of the bleeding, the retrograde wire inserted through the cannula allowed the use of an additional closing device (Perclose ProGlide^®^, Abbott^®^, Chicago, Illinois, United States and/or Angio-Seal^®^, Terumo^®^, Shibuya, Tokyo, Japan), while the retrograde wire (crossover) allowed balloon angioplasty or covered stent implantation. After complete hemostasis, the guidewires were removed, the knots were tightened again, and the reperfusion and contralateral access sites were closed with 6- and 8-Fr Angio-Seal^®^ devices, respectively (Terumo^®^ Shibuya, Tokyo, Japan).

For the removal of the venous cannula, the latter was pulled out and the knots were tightened. In case of persistent bleeding, a figure-of-eight-suture was performed to complete the hemostasis.

### Outcomes

Access-related complications included vascular complications defined as peri-procedural bleeding at the sites of cannulation or the sites of puncture with a drop in hemoglobin level of at least 3.0 g/dL and/or requiring transfusion (3 units of packed red blood cells) [20], acute lower limb ischemia, arterial or vein thrombosis requiring percutaneous or surgical intervention and arterial dissection, and, groin infection. These access related complications were recorded and separated between complications during implantation and support with ECMO, and complications after explantation.

Percutaneous decannulation technical success was defined as successful arterial and vein closure without access-related complications or necessity of adjunctive surgical or endovascular procedures.

### Statistical analyses

Quantitative variables are reported as median (minimum–maximum). Qualitative variables are presented as absolute value and percentage. Continuous variables were compared using Mann–Whitney test. Significance was defined as *p* < 0.05. Excel software was used for all data analyses.

## Results

### Population

**Fig. 1:**
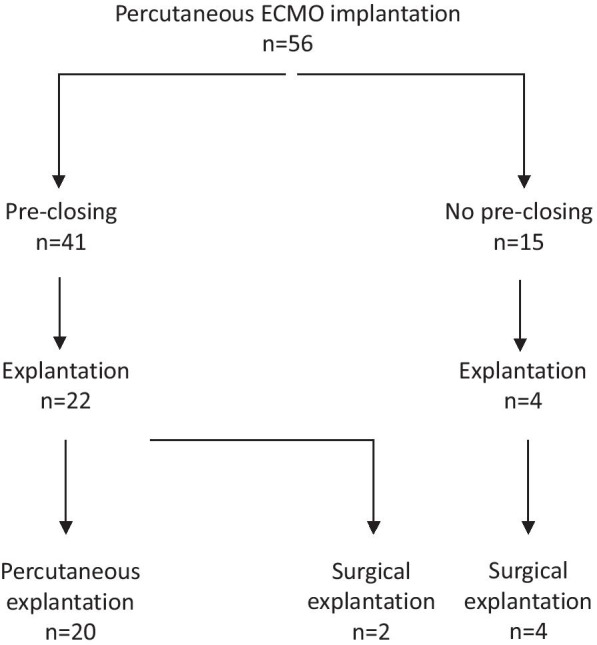
Flowchart of patients with percutaneous implantation of ECMO according to the presence of pre-closing

Between March 2018 and December 2020, 56 patients were treated with percutaneous VA-ECMO in the cardiac catheterization laboratory for refractory cardiogenic shock or cardiac arrest. Among these 56 patients, in 41 preclosing was performed (Flowchart in Fig. 1). The baseline characteristics of these 41 patients of are presented in Table 1.

**Table 1:** Patients’ with preclosing baseline and procedural characteristics

Baseline characteristics	*n* = 41
Age (years)	61 (27–76)
Male [*n* (%)]	30
Weight (kg)	71 (52–110)
Height (cm)	172 (152–192)
BMI (kg/m^2^)	25.1 (17.9–35.9)
Atherosclerosis risk factors	
Tobacco use [*n* (%)]	9 (22)
Arterial hypertension [*n* (%)]	21 (51)
Diabetes mellitus [*n* (%)]	15 (36)
Peripheral artery disease [*n* (%)]	3 (7)
Chronic heart failure [*n* (%)]	4 (10)
Long-term anticoagulation before VA-ECMO [*n* (%)]	3 (7)
Antiplatelet therapy before VA-ECMO [*n* (%)]	8 (19)
Admission	
Cardiac arrest [*n* (%)]	24 (58)
Refractory cardiac arrest (*n*)	5
No flow duration (min)	1 (0–1)
Low flow duration (min)	55 (30–70)
Resuscitated cardiac arrest (n)	19
No flow duration (min)	1 (0–15)
Low flow duration (min)	15 (1–55)
STEMI [*n* (%)]	23 (56)
Associated PCI [*n* (%)]	18 (43)
LVEF (%)^a^	15 (5–55)
SOFA score	10 (3–15)
Procedural characteristics	
Success of implantation [*n* (%)] (%)	41 (100)
Reperfusion cannula [*n* (%)]	40/41 (97)
Cannulation time (min)	20 (10–40)

Continuous data presented as median (min–max)

*BMI* body mass index, *VA-ECMO* veno-arterial extracorporeal membrane oxygenation, *STEMI* ST-elevation myocardial infarction, *PCI* percutaneous coronary intervention, *LVEF* left ventricle ejection fraction

^a^In patients with spontaneous circulation

The 15 patients with no preclosing performed were the first patients with refractory cardiac arrest or severe circulatory failure and ECMO already primed in whom we feared that preclosing might delay ECMO initiation. The baseline, procedural characteristics, and outcomes of the whole population and of patients without preclosing are presented in Additional file 1: Table S1.

The median age of the 41 patients with preclosing was 61 years (27–76) and 73% were male (*n* = 30). Fifteen had diabetes mellitus (36.5%) and 21 had a history of hypertension (51.2%). Of the 41 patients, 5 were referred for refractory cardiac arrest and 36 for cardiogenic shock (following successfully resuscitated cardiac arrest in 19). In patients with successfully resuscitated cardiac arrest, the durations of no flow and low flow were 1 (0–15) minutes and 15 (1–55) minutes, respectively, while in patients with refractory cardiac arrest, they were 1 (0–1) minutes and 55 (30–70) minutes respectively. Of the 41 patients, 18 patients underwent attempted percutaneous coronary intervention. The etiology of cardiogenic shock or cardiac arrest was myocardial infarction (*n* = 23), decompensation of a pre-existing cardiac disease (*n* = 9), fulminant myocarditis (*n* = 4), aortic dissection (*n* = 2), pulmonary embolism (*n* = 1), Tako-Tsubo syndrome (*n* = 1) and of unknown cause (*n* = 1). In patients with spontaneous circulation, the LVEF at the time of ECMO implantation was 15% (5–55). Admission Sequential Organ Failure Assessment (SOFA) score was 10 (3–15).

### ECMO implantation and outcomes

Both arterial and venous cannula were successfully implanted in all 41 patients (Table 1). Distal perfusion line was implanted in 40 patients. Preclosing was successfully performed in all 41 patients (Fig. 1).

Cannulation time was 20 min (10–40) and was not significantly different among patients with or without preclosing (p = 0.73).

Twenty-two of the 41 patients (54%) were weaned from ECMO support at 6 days (2–19) and 12 patients were alive at day 30 (29% of all patients, 60% of patients weaned from ECMO) (Table 2). A major vascular complication related to ECMO implantation occurred in 2 out of 41 patients and was related to distal perfusion line in both (limb ischemia related to self-removal of the reperfusion line and one hemorrhage related to failure of distal perfusion line insertion).

**Table 2:** Patients’ 30-day outcomes

Patient outcomes at 30 days	*n* = 41
30-day mortality (*n*) (%)	30 (71)
ICU stay (days)	13 (1–52)
Weaning from VA-ECMO (*n*) (%)	22 (54)
ECMO duration (days)	5 (1–21)
Percutaneous explantation (*n*)	20
Significant complications (*n*)	2
Significant bleeding (*n*)	1
Acute lower limb ischemia (*n*)	1
Groin infection (*n*)	1 (following surgical explantation)

Continuous data presented as median (min–max)

*ICU* intensive care unit, *VA-ECMO* veno-arterial extracorporeal membrane oxygenation

### Percutaneous explantation and complete percutaneous approach

**Fig. 2:**
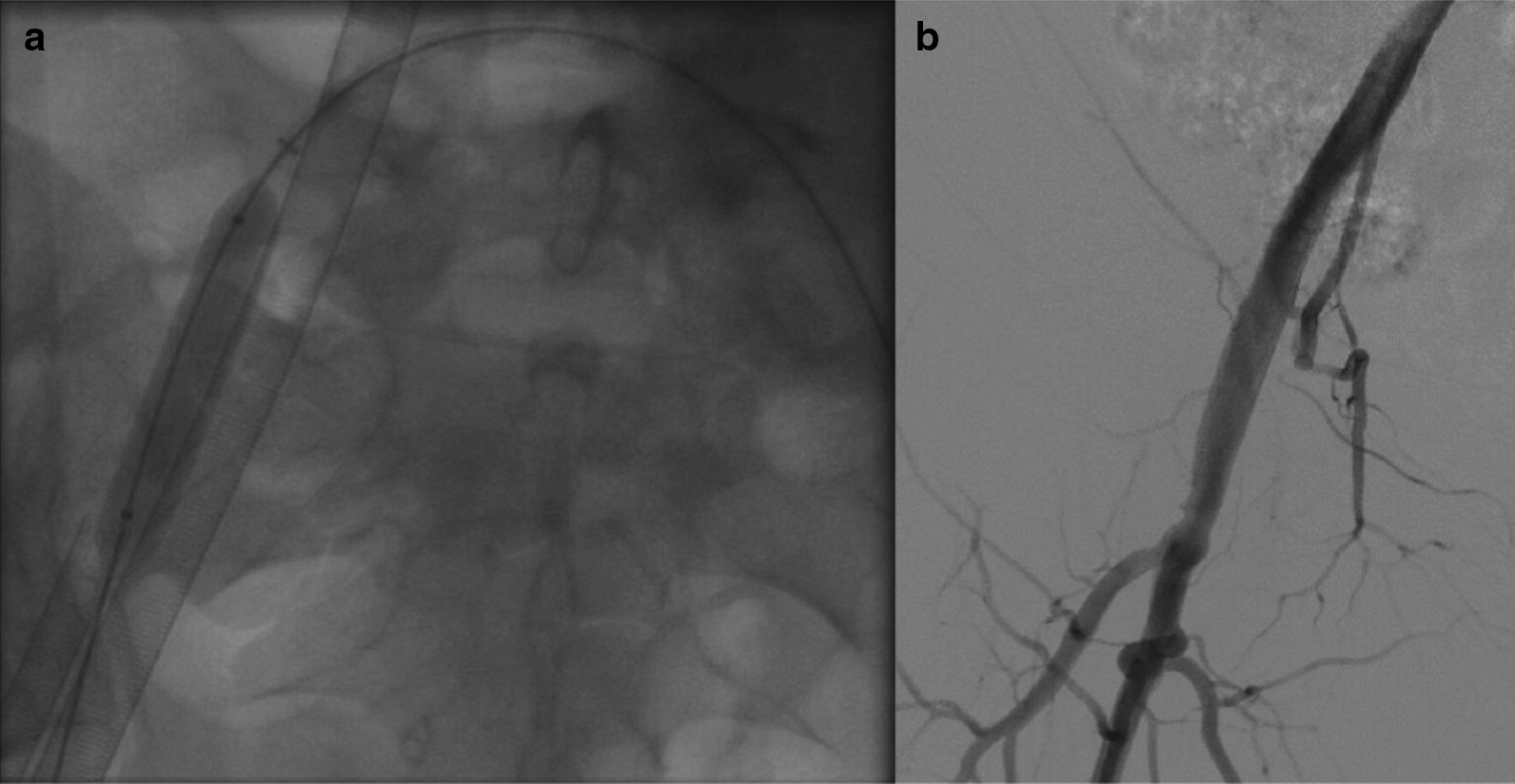
**a** Cross-over balloon technique for arterial cannula safe removal. **b** Final angiography revealing neither residual bleeding nor vascular complication

Among the 41 patients, percutaneous decannulation was performed in 20 patients at 6 days (2–16) (Fig. 1) (Table 3). The “crossover balloon occlusion technique” was used in all 20 patients. Representative angiographies of the closure are presented in Fig. 2. The fluoroscopy time was 6.5 min (2.1–19.6). The contrast volume, dose area product and cumulated air karma were respectively 25 mL (10–140), 1236 cGy/cm^2^ (249–4277) and 140 mGy (23–423) respectively. Arterial closure was successful in all patients except one, in whom surgical closure was needed because of incomplete knots progression toward the arteriotomy. The angiographic control confirmed the closure success without any residual bleeding in the 19 remaining patients and did not reveal any occlusion or dissection of the femoral artery. Venous closure was successful in all patients. One arterio-venous fistula at the site of reperfusion line insertion was diagnosed but did not require surgery.

**Table 3:** Baseline, procedural characteristics and 30-day outcomes of patients with percutaneous decannulation

Percutaneous decannulation	*n* = 20
Patient's characteristics	
Age (years)	60 (27–76)
Cardiac arrest (*n*)	11
Ressuscitated cardiac arrest	10
Refractory cardiac arrest	1
STEMI (n)	13
LVEF (%)^a^	15 (5–45)
SOFA score	8 (3–14)
ECMO duration (days)	6 (2–16)
Decannulation procedure	
Cross-over (*n*)	17
Technical success (*n*)	19
Need of covered stent implantation for hemostasis (*n*)	0 (0)
Fluoroscopy time (min)	6.5 (2.1–19.6)
Iodinated contrast volume (mL)	25 (10–140)
Dose area product (cGy/cm^2^)	1236 (249–4277)
Cumulated air karma (mGy)	140 (23–423)
Outcomes	
30-day mortality (*n*) (%)	11 (55%)
ICU stay (days)	21 (5–47)
Significant vascular complications (n)	1
Need for surgical closure (n)	1
Acute lower limb ischemia (n)	0
Significant bleeding (n)	0
Groin infection (*n*)	0

Continuous data presented as median (min–max)

*STEMI* ST-elevation myocardial infarction, *LVEF* left ventricle ejection fraction, *ECMO* extracorporeal membrane oxygenation, *ICU* intensive care unit

^a^In patients with spontaneous circulation

**Fig. 3:**
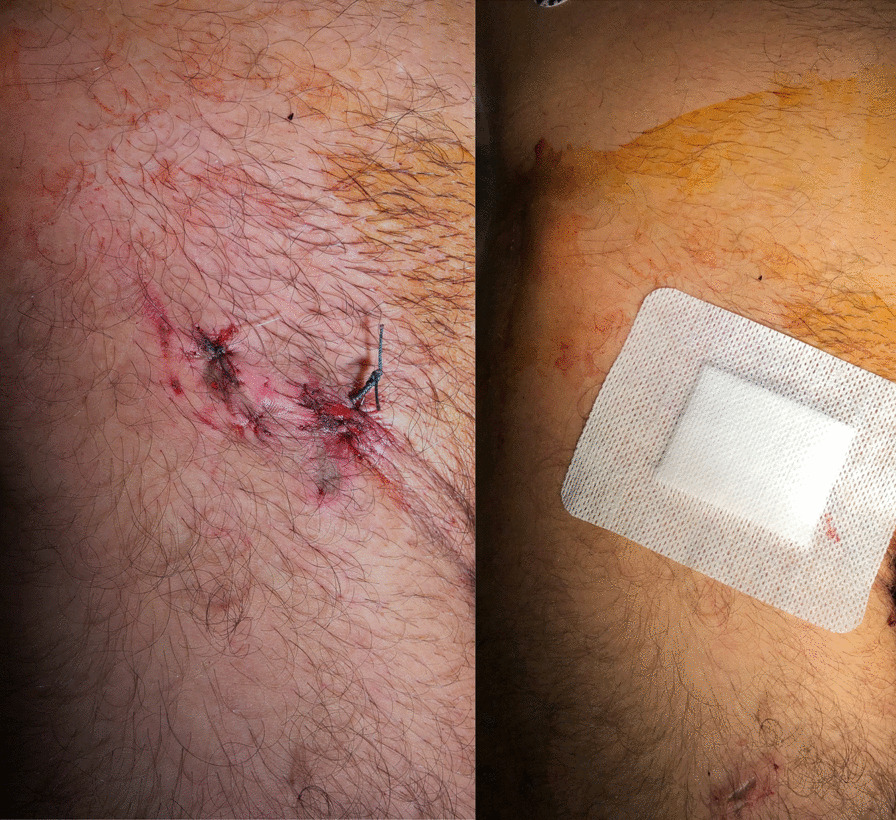
Picture of a patient’s groins after ECMO percutaneous explantation. No additional compression dressing is needed

Then, the skin incision at the puncture site was sutured and no compression dressing was needed (Fig. 3). No vascular complication occurred at the puncture site used for crossover access which was successfully closed in all patients. Finally, no groin wound infection occurred in this subgroup of patients with percutaneous decannulation.

In total, among the 20 patients with complete percutaneous implantation and explantation, only one major complication occurred over the course of ECMO implantation, support and explantation.

Two patients with preclosing had the cannula surgically removed. Those two patients had cardiac surgery for heart transplant and left ventricular assist device implantation, respectively, and the cardiac surgeon decided to surgically explant the ECMO in the operative room following heart surgery, without percutaneous attempt. One of those two patients suffered from a groin wound infection, which occurred after surgical decannulation.

## Discussion

The present study describes the feasibility and safety of a complete angio-guided percutaneous approach for ECMO implantation and explantation in patients with refractory cardiogenic shock or cardiac arrest. The salient findings of this study are the following: (1) angio-guided percutaneous implantation of ECMO is associated with a 100% success of vessel cannulation and a low incidence of vascular complications; (2) percutaneous angio-guided withdrawal of the cannula after preclosing technique provides a good closure of the puncture site and enables a direct confirmation of the result; (3) all in all, this complete percutaneous approach provides an attractive strategy for the management of patients requiring ECMO support with a low risk of vascular complication even in a very high risk population.

Percutaneous implantation of VA-ECMO has been previously described and one large retrospective registry suggested that it was associated with fewer complications than surgical approach [10, 11]. However, to date, there are only sparse data regarding angio-guided percutaneous implantation in the catheterization laboratory although insertion in the catheterization room is becoming standard of care if numerous centers. Indeed, in patients with cardiogenic shock or cardiac arrest related to acute coronary syndrome or requiring urgent percutaneous coronary intervention, the respective timing of ECMO implantation and percutaneous coronary intervention is controversial [21–23] and percutaneous implantation in the catheterization laboratory allows to quickly move from one strategy to the other. In our study, percutaneous cannulation was successful in all patients with a very low incidence of vascular complications. The previous studies that had described percutaneous ECMO implantation in the catheterization laboratory had reported much higher rates of vascular complications as compared to our study (20–40% vs. 2/41) [24]. This finding is likely to be at least partially related to the absence of systematic insertion of a distal perfusion line, which was left at the operator’s choice in those studies although this has been associated to a decrease rate of complications [25, 26]. These encouraging results regarding ECMO implantation were not altered by the performance of preclosing. In patients with preclosing, the number of major complication during ECMO was small (2/41). Also, it is important to highlight that the cannulation time was not increased by the performance of pre-closing. Indeed, when mastered, the insertion of all ProGlide devices for preclosing requires less than one minute of time and does not delay ECMO initiation.

Regarding ECMO explantation, different strategies have been described for cannula removal after percutaneous implantation. In the largest cohort reported so far, hemostasis was made using compression only, with 45 min of manual compression followed by the use of the FemoStop^®^ compression assist device (Abbott^®^, Chicago, Illinois, United States) [11]. Although easy and convenient, this technique was associated with a 15% rate of vascular complication requiring surgical revision after cannula removal. Few closure devices have been tested for VA ECMO decannulation. The Manta^®^ vascular closure device (VCD^®^, Teleflex, Morrisville, USA) has been used in several case reports and in a cohort of 16 patients with good results regarding hemostasis but with a rather high rate of arterial stenosis or thrombosis following vascular closure (27.3%) [13]. The Perclose ProGlide^®^ closure device has been studied for post-closing with a rate of failure between 5 and 15% and the use of 2 to 4 devices [14, 15]. This technique is attractive although in large bore vascular access, the needles of the Perclose ProGlide^®^ sometimes do not perforate the vessel wall, making percutaneous closure impossible. The preclosing technique using the Perclose ProGlide^®^ devices is therefore attractive. Since the closure devices are inserted before the large sheat, it is very unlikely that the needle will not properly perforate the vessel wall. Moreover, with the increase of structural heart disease interventions and large bore vascular accesses, this technique is now mastered by most of the interventional cardiologists. Preclosing has been studied by Pellenc et al. in ECMO implanted prior to lung transplantation, with a dramatic reduction in the rate of groin wound infection as compared to open surgery [12]. The patients included in this study were however different from ours. First, they all underwent ECMO implantation in the setting of scheduled lung transplantation while, in our study, all ECMO were implanted in the context of extreme emergency. Second, the diameter of the arterial cannula was 15 Fr compared to 17–19 Fr in our patients. Despite these differences, the rate of vascular complication after explantation was not higher in our study. This is probably related to the use of the “crossover balloon occlusion technique”. This technique, which has been described for TAVR, allows for a safe and smooth closure of the artery, since the upstream vessel is occluded by a balloon [17]. Moreover, after closure, an arteriography is performed to ensure the proper closure of the vessel, without bleeding, or downstream occlusion or dissection. This final angiography enables a quick diagnosis of any remaining bleeding after closure (even small), with the purpose that an additional closure device can be immediately added if necessary.

An additional, although small, advantage of preclosing before ECMO insertion is the treatment of bleeding or suffusion around the cannula. Indeed, the preclosing devices are creating a purse-string around the cannula. Thus, in case of bleeding at the puncture site after cannula insertion, the sutures of the closing devices can be tightened by gently pulling the rail suture until no residual bleeding is observed [27].

Finally, we did not observe any groin infection after complete percutaneous implantation and explantation of ECMO. Percutaneous implantation of ECMO has been previously associated with a lower risk of infection but few studies were focused on a complete percutaneous approach, all the more in the setting of emergent implantation. It is likely that, as previously described in scheduled patients, our results regarding infection are to some extend related to the complete percutaneous approach, although the absence of a control group do not allow any definitive conclusion.

These encouraging results regarding both percutaneous implantation and explantation of ECMO in these patients with refractory cardiogenic shock may encourage its early use in such patients in whom early restoration of an adequate organ perfusion is essential for favorable outcome.

## Limitations

This study carries several limitations. First, this is a monocentric study including a small number of patients. Hence, larger studies including a control group are needed to confirm our results. Second, all ECMO implantations were performed in extreme emergency and a significant proportion of them were implanted outside working hours by the on call interventional cardiologist. This requires all interventional cardiologists of the department to be trained to preclosing and large bore vascular access. Moreover, in our study, this technique was safe and feasible up to 16 days post-ECMO implantation. None of our patients had percutaneous decannulation after day 16 and we do not know whether it remains safe beyond this period. Last, the studied population was composed of dramatically instable patients with a high mortality rate albeit consistent with other studies focusing on refractory cardiac arrest and cardiogenic shock [9, 24] and very few patients with refractory cardiac arrest underwent ECMO explantation. However, despite this very high risk, few complications occurred and almost half of the patients were weaned from ECMO, suggesting that the results of complete percutaneous ECMO may be even better in a less unstable population.

## Conclusion

Emergent complete percutaneous angio-guided VA-ECMO implantation and explantation using preclosing technique is an attractive strategy in patients referred for refractory cardiogenic shock with few major complications. These results encourage the early use of percutaneous ECMO in patients referred for cardiogenic shock.

## Supplementary Information

**Additional file 1. Table S1**: Whole population and patients without preclosing baseline characteristics, procedural characteristics and outcomes.

## Data Availability

The datasets used and/or analyzed during the current study are available from the corresponding author on reasonable request.

## References

[1] Reynolds HR, Hochman JS (2008). Cardiogenic shock: current concepts and improving outcomes. Circulation.

[2] Topalian S, Ginsberg F, Parrillo JE (2008). Cardiogenic shock. Crit Care Med.

[3] Gallet R, Lellouche N, Mitchell-Heggs L, Bouhemad B, Bensaid A, Dubois-Randé J-L (2012). Prognosis value of central venous oxygen saturation in acute decompensated heart failure. Arch Cardiovasc Dis.

[4] Levy B, Clere-Jehl R, Legras A, Morichau-Beauchant T, Leone M, Frederique G (2018). Epinephrine versus norepinephrine for cardiogenic shock after acute myocardial infarction. J Am Coll Cardiol.

[5] Mebazaa A, Combes A, van Diepen S, Hollinger A, Katz JN, Landoni G (2018). Management of cardiogenic shock complicating myocardial infarction. Intensive Care Med.

[6] Ponikowski P, Voors AA, Anker SD, Bueno H, Cleland JGF, Coats AJS (2016). ESC Guidelines for the diagnosis and treatment of acute and chronic heart failure: the task force for the diagnosis and treatment of acute and chronic heart failure of the European Society of Cardiology (ESC) developed with the special contribution of the Heart Failure Association (HFA) of the ESC. Eur Heart J.

[7] Combes A, Leprince P, Luyt C-E, Bonnet N, Trouillet J-L, Léger P (2008). Outcomes and long-term quality-of-life of patients supported by extracorporeal membrane oxygenation for refractory cardiogenic shock. Crit Care Med.

[8] Han KS, Kim SJ, Lee EJ, Jung JS, Park JH, Lee SW (2019). Experience of extracorporeal cardiopulmonary resuscitation in a refractory cardiac arrest patient at the emergency department. Clin Cardiol.

[9] Pozzi M, Armoiry X, Achana F, Koffel C, Pavlakovic I, Lavigne F (2020). Extracorporeal life support for refractory cardiac arrest: a 10-year comparative analysis. Ann Thorac Surg.

[10] Ganslmeier P, Philipp A, Rupprecht L, Diez C, Arlt M, Mueller T (2011). Percutaneous cannulation for extracorporeal life support. Thorac Cardiovasc Surg.

[11] Danial P, Hajage D, Nguyen LS, Mastroianni C, Demondion P, Schmidt M (2018). Percutaneous versus surgical femoro-femoral veno-arterial ECMO: a propensity score matched study. Intensive Care Med.

[12] Pellenc Q, Girault A, Roussel A, Aguir S, Cerceau P, Longrois D (2020). Preclosing of the femoral artery allows total percutaneous venoarterial extracorporeal membrane oxygenation and prevents groin wound infection after lung transplantation. Eur J Cardio-Thorac Surg Off J Eur Assoc Cardio-Thorac Surg.

[13] Bemtgen X, Heidt T, Zotzmann V, Rilinger J, Wengenmayer T, Biever PM (2020). Venoarterial extracorporeal membrane oxygenation decannulation using the novel Manta vascular closure device. Eur Heart J Acute Cardiovasc Care.

[14] Hwang J (2016). Percutaneous removal using Perclose ProGlide closure devices versus surgical removal for weaning after percutaneous cannulation for venoarterial extracorporeal membrane oxygenation. J Vasc Surg.

[15] Lüsebrink E, Stremmel C, Stark K, Petzold T, Hein-Rothweiler R, Scherer C (2019). Percutaneous decannulation instead of surgical removal for weaning after venoarterial extracorporeal membrane oxygenation—a crossed perclose proglide closure device technique using a hemostasis valve Y connector. Crit Care Explor.

[16] Torsello GB, Kasprzak B, Klenk E, Tessarek J, Osada N, Torsello GF (2003). Endovascular suture versus cutdown for endovascular aneurysm repair: a prospective randomized pilot study. J Vasc Surg.

[17] Genereux P, Kodali S, Leon MB, Smith CR, Ben-Gal Y, Kirtane AJ (2011). Clinical outcomes using a new crossover balloon occlusion technique for percutaneous closure after transfemoral aortic valve implantation. JACC Cardiovasc Interv.

[18] Griese DP, Reents W, Diegeler A, Kerber S, Babin-Ebell J (2013). Simple, effective and safe vascular access site closure with the double-ProGlide preclose technique in 162 patients receiving transfemoral transcatheter aortic valve implantation. Catheter Cardiovasc Interv Off J Soc Card Angiogr Interv.

[19] Yoshimura N, Ataka K, Nakagiri K, Azami T, Yoshida M, Yamashita C (1996). A simple technique for the prevention of lower limb ischemia during femoral veno-arterial cardiopulmonary support. J Cardiovasc Surg (Torino).

[20] Kappetein AP, Head SJ, Généreux P, Piazza N, van Mieghem NM, Blackstone EH (2013). Updated standardized endpoint definitions for transcatheter aortic valve implantation: the Valve Academic Research Consortium-2 consensus document. J Thorac Cardiovasc Surg.

[21] Vallabhajosyula S, Prasad A, Sandhu GS, Bell MR, Gulati R, Eleid MF (2019). Mechanical circulatory support-assisted early percutaneous coronary intervention in acute myocardial infarction with cardiogenic shock: 10-year national temporal trends, predictors and outcomes. Euro Intervent J Eur Collab Work Group Interv Cardiol Eur Soc Cardiol.

[22] Sheu J-J, Tsai T-H, Lee F-Y, Fang H-Y, Sun C-K, Leu S (2010). Early extracorporeal membrane oxygenator-assisted primary percutaneous coronary intervention improved 30-day clinical outcomes in patients with ST-segment elevation myocardial infarction complicated with profound cardiogenic shock. Crit Care Med.

[23] Huang C-C, Hsu J-C, Wu Y-W, Ke S-R, Huang J-H, Chiu K-M (2018). Implementation of extracorporeal membrane oxygenation before primary percutaneous coronary intervention may improve the survival of patients with ST-segment elevation myocardial infarction and refractory cardiogenic shock. Int J Cardiol.

[24] Ternus B, Jentzer J, Bohman K, Barsness G, Schears G, Rihal C (2020). Initiation of extracorporeal membrane oxygenation in the cardiac catheterization laboratory: the mayo clinic experience. J Invasive Cardiol.

[25] Lamb KM, DiMuzio PJ, Johnson A, Batista P, Moudgill N, McCullough M (2017). Arterial protocol including prophylactic distal perfusion catheter decreases limb ischemia complications in patients undergoing extracorporeal membrane oxygenation. J Vasc Surg.

[26] Jia D, Yang IX, Ling RR, Syn N, Poon WH, Murughan K (2020). Vascular complications of extracorporeal membrane oxygenation: a systematic review and meta-regression analysis. Crit Care Med.

[27] Melloni A, Grandi A, Melissano G, Chiesa R, Bertoglio L (2020). Safety and feasibility of percutaneous purse-string-like downsizing for femoral access during complex endovascular aortic repair. Cardiovasc Intervent Radiol.

