# Supplementary material for: Complete percutaneous angio-guided approach using preclosing for venoarterial extracorporeal membrane oxygenation implantation and explantation in patients with refractory cardiogenic shock or cardiac arrest
Source: Crit Care. 2021 Mar 7;25:93. doi: 10.1186/s13054-021-03522-8 (PMC7938494; doi:10.1186/s13054-021-03522-8)
Supplement: Supplementary file 1 — Additional file 1. Table S1: Whole population and patients without preclosing baseline characteristics, procedural characteristics and outcomes. [file 13054_2021_3522_MOESM1_ESM.docx]

**Additional table 1: Whole population and patients without preclosing baseline characteristics, procedural characteristics and outcomes**

| **Baseline characteristics** | **All patients**  **N=56** | **No pre-closing N=15** |
| --- | --- | --- |
| Age (years) | 61 (27-84) | 63 (44-84) |
| Male (n) | 42 | 12 |
| Weight (Kg) | 72 (52-130) | 77 (60-130) |
| Height (cm) | 171 (143-192) | 170 (143-181) |
| BMI (kg/m²) | 25.4 (17.9-40.1) | 26.1 (22.9-40.1) |
| **Atherosclerosis risk factors** |  |  |
| Tobacco use (n) | 11 | 2 |
| Arterial hypertension (n) | 32 | 11 |
| Diabetes mellitus (n) | 22 | 7 |
| Peripheral artery disease (n) | 3 | 0 |
| Chronic heart failure (n) | 6 | 2 |
| Long-term anticoagulation before VA-ECMO (n) | 4 | 1 |
| Antiplatelet therapy before VA-ECMO (n) | 12 | 4 |
| **Admission** |  |  |
| Cardiac arrest (n) | 36 | 12 |
| Refractory cardiac arrest n   - No flow duration (min) - Low flow duration (min) | 15  <1 (0-5)  51 (23-111) | 10  <1 (0-5)  47 (23-111) |
| Resuscitated cardiac arrest :   - No flow duration (min) - Low flow duration (min) | 21  <1 (0-15)  15 (1-55) | 2  1 (0-2)  12 (5-20) |
| STEMI (n) | 34 | 11 |
| Associated PCI (n) | 29 | 11 |
| LVEF (%)***^1^*** | 15 (5-55) | 20 (15-50) |
| SOFA score | 10 (3-15) | 11 (6-14) |
| **Procedural characteristics** |  |  |
| Success of implantation (n) (%) | 56 (100) | 15 (100) |
| Reperfusion cannula (n) | 54/56 | 14/15 |
| Cannulation time (min) | 20 (10-40) | 25 (10-30) |
| **Patient outcomes at 30 days** |  |  |
| 30-day mortality (n) (%) | 43 (77) | 13 (87) |
| ICU stay (days) | 10 (1-85) | 2 (1-85) |
| Weaning from VA-ECMO (n) (%) | 26 (46) | 4 (26) |
| ECMO duration (days) | 4.5 (1-40) | 1.0 (1-40) |

***^1^*** *In patients with spontaneous circulation*

*BMI: Body mass index; VA-ECMO: Veno-arterial extracorporeal membrane oxygenation; STEMI: ST-elevation myocardial infarction; PCI: percutaneous coronary intervention; LVEF: Left Ventricle ejection fraction; ICU: Intensive care unit.*

*Continuous data presented as median (min-max)*
